# Analgesic Mechanisms of Steroid Ointment against Oral Ulcerative Mucositis in a Rat Model

**DOI:** 10.3390/ijms222212600

**Published:** 2021-11-22

**Authors:** Mako Naniwa, Chihiro Nakatomi, Suzuro Hitomi, Kazunari Matsuda, Takuya Tabuchi, Daijiro Sugiyama, Sayaka Kubo, Yuichi Miyamura, Kenichi Yoshino, Sumio Akifusa, Kentaro Ono

**Affiliations:** 1 Division of Physiology, Kyushu Dental University, 2-6-1 Manazuru, Kokurakita-ku, Kitakyushu 803-8580, Fukuoka, Japan; r16naniwa@fa.kyu-dent.ac.jp (M.N.); r20nakatomi@fa.kyu-dent.ac.jp (C.N.); hitomi.suzuro@nihon-u.ac.jp (S.H.); r16miyamura@fa.kyu-dent.ac.jp (Y.M.); 2 School of Oral Health Sciences, Kyushu Dental University, 2-6-1 Manazuru, Kokurakita-ku, Kitakyushu 803-8580, Fukuoka, Japan; r11akifusa@fa.kyu-dent.ac.jp; 3 R&D Department, Daiichi Sankyo Healthcare Co., Ltd., 1-2-58 Hiromachi, Shinagawa-ku, Tokyo 140-8710, Japan; matsuda.kazunari.x7@daiichisankyo-hc.co.jp (K.M.); tabuchi.takuya.td@daiichisankyo-hc.co.jp (T.T.); sugiyama.daijiro.gz@daiichisankyo-hc.co.jp (D.S.); kubo.sayaka.hr@daiichisankyo-hc.co.jp (S.K.); 4 Section of Primary Dental Education, Kyushu Dental University, 2-6-1 Manazuru, Kokurakita-ku, Kitakyushu 803-8580, Fukuoka, Japan; kyoshino@kyu-dent.ac.jp

**Keywords:** stomatitis, triamcinolone acetonide, glucocorticoids, pain, orofacial pain, trigeminal ganglion, drug delivery systems

## Abstract

Despite the long history of use of steroid ointments for oral mucositis, the analgesic mechanism has not been fully elucidated. In this study, we examined the effects of triamcinolone acetonide (Tmc) on oral ulcerative mucositis-induced pain in conscious rats by our proprietary assay system. Based on evaluations of the physical properties and retention periods in the oral mucosa of human volunteers and rats, we selected TRAFUL^®^ ointment as a long-lasting base. In oral ulcerative mucositis model rats, TRAFUL^®^ with Tmc suppressed cyclooxygenase-dependent inflammatory responses with upregulations of glucocorticoid receptor-induced anti-inflammatory genes and inhibited spontaneous nociceptive behavior. When an ointment with a shorter residual period was used, the effects of Tmc were not elicited or were induced to a lesser extent. Importantly, TRAFUL^®^ with Tmc also improved oral ulcerative mucositis-induced mechanical allodynia, which has been reported to be independent of cyclooxygenase. Ca^2+^ imaging in dissociated trigeminal ganglion neurons showed that long-term preincubation with Tmc inhibited the hypertonic stimulation-induced Ca^2+^ response. These results suggest that the representative steroid Tmc suppresses oral ulcerative mucositis-induced pain by general anti-inflammatory actions and inhibits mechanical sensitivity in peripheral nerves. For drug delivery, long-lasting ointments such as TRAFUL^®^ are needed to sufficiently induce the therapeutic effects.

## 1. Introduction

Oral ulcer can be a frequent complication of oral mucositis [1]. In addition to recurrent aphthous stomatitis, oral ulcerative mucositis develops by biting and mechanical trauma from dentures and orthodontic appliances [2,3]. In many cancer patients receiving chemoradiotherapy, severe ulcerative mucositis occurs in the oral cavity [4,5,6]. Oral ulcerative mucositis elicits severe pain during meals and speaking, ultimately resulting in poor quality of life [7,8,9]. Steroid ointments are generally used for the treatment of oral ulcerative mucositis. The representative steroid used for oral ulcerative mucositis is triamcinolone acetonide (Tmc), a synthetic glucocorticoid [10,11,12]. Steroidal drugs activate intracellular glucocorticoid receptors, which in the nucleus upregulate the expression of various target genes that suppress the nuclear factor kappa B (NF-κB) pathway, resulting in anti-inflammatory effects via the suppression of cyclooxygenase-2 (COX-2) and tumor necrosis factor-α (TNF-α) production [13,14].

Recent animal studies using rat models of oral ulcerative mucositis have suggested that oral ulcerative mucositis lead to two different pain modalities: COX-dependent spontaneous pain and COX-independent mechanical allodynia [15,16]. Therefore, the well-known steroid-induced therapeutic effect is expected to suppress COX-dependent pain in oral ulcerative mucositis, but not COX-independent mechanical allodynia. Clinically, there is no consensus as to whether steroid ointments exert analgesic effects [17,18,19,20,21]. Similarly, there is little agreement about wound healing with this treatment [17,18,19,20,21]. Some studies have reported delays in healing following systemic administration of other corticosteroids [22,23] and local treatment [24]. Relative to systemic administration, region-specific local treatment with ointments has fewer side effects [25]; in the case of steroid administration, hyperglycemia and inhibition of systemic immune responses often occur [26].

In the present study, to examine the analgesic mechanism of steroids in oral ointments, we investigated the effects of Tmc on oral ulcerative mucositis-induced pain in a rat model by our proprietary assay system for conscious rats [15]. Plastibase hydrocarbon gel is widely used for oral ointment bases. However, this commonly used ointment base has a residual period on the oral mucosa of conscious rats that was too short to demonstrate clear glucocorticoid efficacy. Therefore, we first explored long-lasting ointment bases through physical, human, and animal tests and subsequently investigated the analgesic mechanism of steroids for nociception in oral ulcerative mucositis model (OUM) rats.

## 2. Results

### 2.1. Residual Property of Ointment Bases on the Oral Mucosa

In the analysis of the physical properties of the four kinds of ointment bases, TRAFUL^®^ base (Traful) and TRAFUL^®^ PRO-quick base (PRO-quick) had significantly higher adhesiveness, hardness, and viscosities at two different rotational speeds (1/s = 1 and 10) than Vaseline and Plastibase (Table 1).

**Figure 1:**
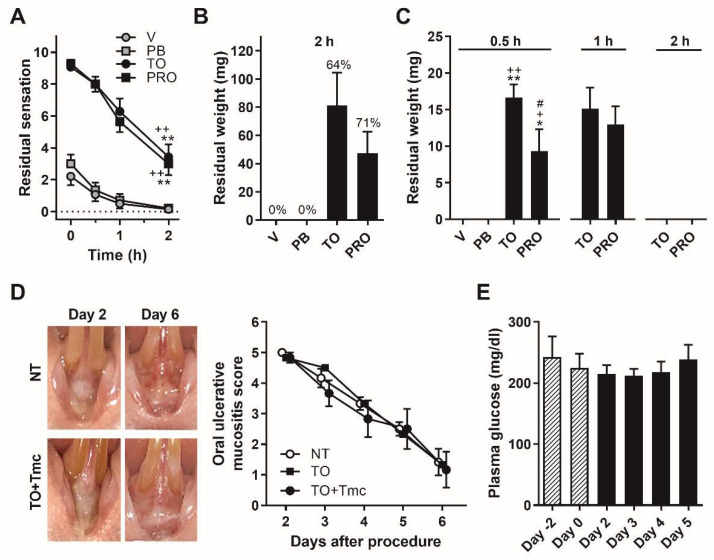
Residual periods of ointments on the oral mucosa of healthy humans and rats and the effects of triamcinolone acetonide (Tmc) on the oral ulcerative mucositis score and blood glucose level. V: Vaseline, PB: Plastibase, TO: Traful and PRO: Traful PRO-quick. (**A**) Residual sensation of each ointment at four different mucosal areas on the back of the upper and lower lip. The ointments were randomly applied by the participant’s fingers (*n* = 14). ** and ^++^, *p* < 0.01 for all time points, compared with V and PB, respectively, according to Tukey’s test following two-way ANOVA. (**B**) Residual weights of ointments on the oral mucosa of healthy humans 2 h after application. TO and PRO could be collected from 64% (*n* = 9/14) and 71% (*n* = 10/14) of the subjects. (**C**) Residual weights of ointments collected from the labial fornix region of rats (each ointment, *n* = 4). * and ^+^, *p* < 0.05 and ** and ^++^, *p* < 0.01 compared with V and PB, respectively, and ^#^, *p* < 0.05 compared with TO according to Tukey’s test following one-way ANOVA (*f* = 0.89). (**D**) Effects of TO and TO with Tmc on the oral ulcerative mucositis score (two-way ANOVA, *f* = 0.46, 0.74, 0.80, 0.82, 0.86 at days 2–6, respectively) in the oral ulcerative mucositis model. NT: non-treatment, TO: TO treatment without Tmc and TO + Tmc: TO treatment with Tmc; each group *n* = 6. (**E**) Effect of TO with Tmc on blood glucose levels in the oral ulcerative mucositis model (*n* = 5, one-way ANOVA, *f* = 0.25).

In 14 healthy human volunteers without any oral mucositis, the residual sensations of Traful and PRO-quick were higher than those of Vaseline and Plastibase (*p* < 0.01 for all time points, Tukey’s test following two-way ANOVA test; Figure 1A). At 2 h after application, the residual sensations of Traful and PRO-quick remained, but little residual sensation remained for Vaseline and Plastibase. At the 2-h time point, Traful and PRO-quick could actually be collected from 64% (*n* = 9/14) and 71% (*n* = 10/14) of the subjects, respectively, but Vaseline and Plastibase could not be collected (Figure 1B).

In all rats, Traful and PRO-quick could be collected 30 min after application, but Vaseline and Plastibase could not be collected (Traful: *p* < 0.01 compared with Vaseline and Plastibase, PRO-quick: *p* < 0.05, compared with Vaseline, Plastibase and Traful, Tukey’s test following one-way ANOVA test; Figure 1C). Furthermore, Traful and PRO-quick could be collected from all rats at 1 h, although they were not present in the mucosal region at 2 h (Figure 1C).

As Traful showed the highest mean collection amounts among the ointments in humans (Figure 1B) and rats (Figure 1C), we selected Traful as an appropriate oral ointment with long-lasting residual performance for the following animal experiments.

### 2.2. Effects of a Steroid-Containing Ointment on Oral Ulcerative Mucositis Healing and Plasma Glucose Levels in OUM Rats

The rats with oral ulcerative mucositis showed gradual oral ulcerative mucositis healing (nontreated group; Figure 1D), similar to our previous studies [15,27]. Two coats of Traful did not affect the oral ulcerative mucositis healing between days 2 and 6 (Figure 1D), indicating that mechanical injury to the oral ulcer was not induced by the usage of toothpicks. Daily treatment with Tmc-containing Traful did not result in any changes in oral ulcerative mucositis healing or plasma glucose levels (Figure 1D,E). These results indicate that the local treatment with steroid-containing Traful does not elicit the well-known side effects of steroids.

### 2.3. Anti-Inflammatory Effects of a Steroid-Containing Ointment on OUM Rats

**Figure 2:**
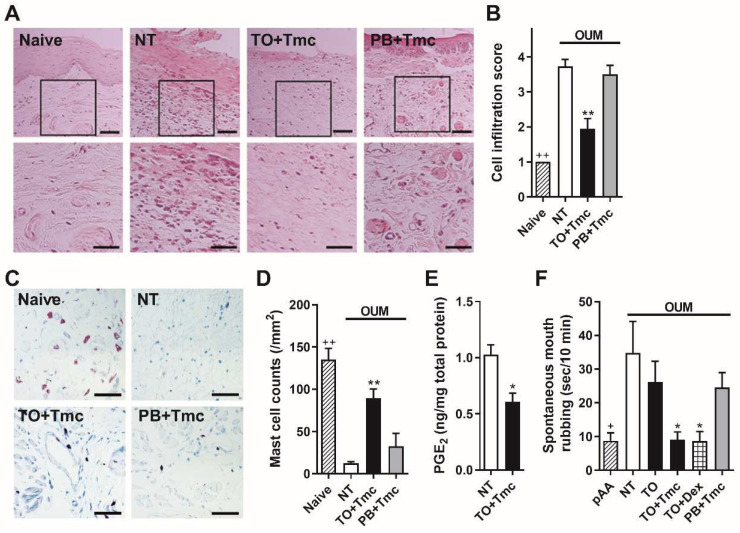
Anti-inflammatory effects of triamcinolone acetonide (Tmc) in Traful ointment (TO) and analgesic effect on spontaneous pain in the oral ulcerative mucositis model (OUM, 2 days after the acetic acid procedure). (**A**) Representative histological images of the oral mucosa in the naive rats and the oral ulcerative mucositis after local treatments with Tmc-containing ointments. NT: non-treatment, TO + Tmc: TO with Tmc and PB + Tmc: Plastibase with Tmc. Microphotographs in the lower panel are enlarged from square area in the microphotographs in the upper panel (scale bars = 50 µm). Oral ulcerative mucositis sections were collected from the OUM following 2 coats of ointments. Tissue sections from the other 2 rats in each group exhibited the same features. (**B**) Semi-quantitative analyses of inflammatory cell infiltration in the oral mucosa (*n* = 3 in each group) using a 4-step score (1: none, 2: mild, 3: moderate and 4: severe). ^++^ *p* < 0.01 between naive and NT according to Student’s *t*-test (*d* = 8.00). Among OUM rats, ** *p* < 0.01, compared with NT according to Sidak’s test following one-way ANOVA (*f* = 0.79) (**C**) Representative toluidine blue-stained images of the oral mucosa in the naive and the OUM rats after local treatments with Tmc-containing ointments. (**D**) Quantitative analyses of mast cell counts in the oral mucosa (*n* = 3 in each group). ^++^ *p* < 0.01 between naive and NT according to Student’s *t*-test (*d* = 7.49). Among OUM rats, ** *p* < 0.01, compared with NT according to Sidak’s test following one-way ANOVA (*f* = 0.85) (**E**) Prostaglandin E_2_ (PGE_2_) levels in the oral ulcerative mucositis in the NT and TO + Tmc rats (each *n* = 4). * *p* < 0.05, compared with NT according to Student’s *t*-test (*d* = 2.55). (**F**) Effects of ointments on spontaneous mouth rubbing. NT (pAA: before the acetic acid procedure in the same rats), TO: TO alone, TO + Tmc, TO + Dex: TO with dexamethasone and PB + Tmc; each group *n* = 5. ^+^ *p* < 0.05 between pAA and NT according to Student’s *t*-test (*d* = 1.45). Among OUM rats, * *p* < 0.05, compared with NT according to Sidak’s test following one-way ANOVA (*f* = 0.20).

The histology of the oral mucosa in the naive rats indicated that the mucosal surface was fully covered by a stratified epithelium that was well keratinized (*n* = 3, Figure 2A). As reported in our previous studies [15,27], OUM rats showed ulceration, inflammatory cell infiltration (*n* = 3, Figure 2A), and mast cell degranulation on day 2 (*n* = 3, Figure 2C). However, treatment with Tmc-containing Traful suppressed inflammatory cell infiltration and mast cell degranulation on the same day, despite the presence of oral ulcerative mucositis (*n* = 3, Figure 2A,C), and suppressed the prostaglandin E_2_ levels in the ulcerative region (*n* = 4, *p* < 0.05 Student’s *t*-test compared with the nontreated group, *n* = 4; Figure 2E). Treatment with Tmc-containing Plastibase did not affect the histology of the oral ulcerative mucositis (*n* = 3, Figure 2A,C). In semi-quantitative analysis, oral ulcerative mucositis demonstrated significant cell infiltration compared with naïve rats (^++^, *p* < 0.01, Student’s *t*-test; Figure 2B). Treatment with Tmc-containing Traful reduced the increment of cell infiltration score in the oral ulcerative mucositis (** *p* < 0.01, compared with the non-treatment group according to Sidak’s test following one-way ANOVA, Figure 2B). Similarly, mast cell density was significantly reduced in the oral ulcerative mucositis, relative to naïve rats (^++^, *p* < 0.01, Student’s *t*-test; Figure 2D). Treatment with Tmc-containing Traful increased the mast cell density (** *p* < 0.01, compared with the non-treatment group according to Sidak’s test following one-way ANOVA, Figure 2D).

Spontaneous mouth rubbing behavior significantly increased at day 2 compared with that before the acetic acid procedure (*n* = 5, *p* < 0.05, Student’s *t*-test; Figure 2F), indicating the development of spontaneous pain. Treatment with Tmc-containing Traful inhibited spontaneous pain (*n* = 5, *p* < 0.05, Sidak’s test following one-way ANOVA), but Traful alone (*n* = 5) did not (Figure 2F). Treatment with Traful containing another steroid, dexamethasone, (*n* = 5), induced the same effects on spontaneous pain as Tmc (*p* < 0.05, Sidak’s test). Treatment with Tmc-containing Plastibase (*n* = 5) did not show any effect (Figure 2F).

**Figure 3:**
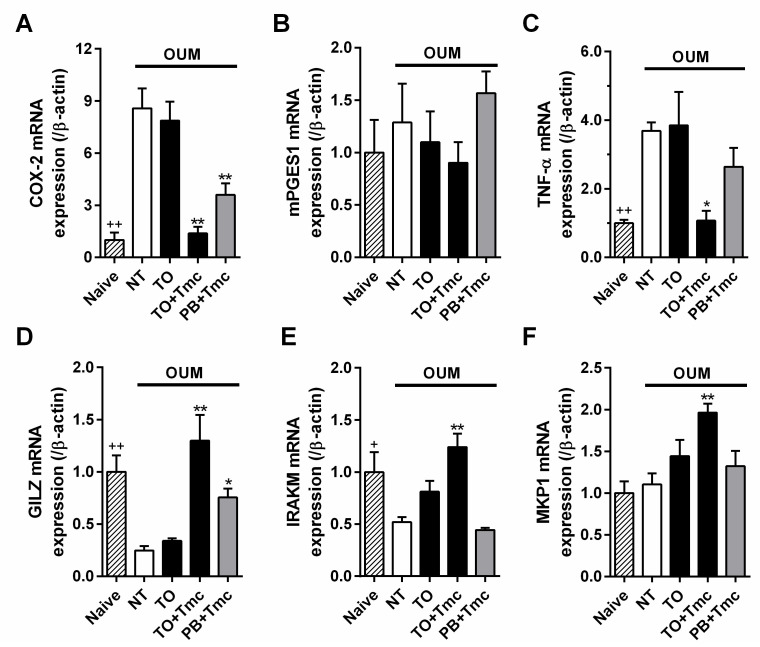
Changes in gene expression following two ointment treatments. ^+^ *p* < 0.05 and ^++^ *p* < 0.01 between the naive rats and the nontreated (NT) oral ulcerative mucositis model (OUM) rats according to Student’s *t*-test. Among OUM rats, * *p* < 0.05 and ** *p* < 0.01, compared with NT according to Sidak’s test following one-way ANOVA. TO: Traful ointment alone; TO + Tmc: TO with triamcinolone acetonide (Tmc); and PB + Tmc: Plastibase with Tmc. For each group, *n* = 5 (*d* = 2.91, 0.17, 6.80, 3.77, 1.19, 0.57. *f* = 0.87, 0.38, 0.74, 0.81, 0.77, and 0.72). (**A**) Cyclooxygenase-2 (COX-2). (**B**) Microsomal prostaglandin E synthase-1 (mPGES1). (**C**) Tumor necrosis factor-α (TNF-α). (**D**) Glucocorticoid-induced leucine zipper (GILZ). (**E**) Interleukin-1 receptor-associated kinases M (IRAKM). (**F**) Mitogen-activated protein kinase phosphatase 1 (MKP1).

To examine the effects of Tmc on mRNA expressions, we investigated inflammatory and anti-inflammatory gene expression levels in the oral mucosa in the naive and model rats on day 2 following two coats of ointment. Compared with that of the naive rats (*n* = 5), the mRNA level of COX-2 was significantly upregulated in the model rats (*p* < 0.01, Student’s *t*-test; Figure 3A), while the mRNA level of microsomal prostaglandin E synthase-1 (mPGES1) was not changed (Figure 3B). Similarly, the mRNA level of the TNF-α was also upregulated (*p* < 0.01, compared with the naive rats, Student’s *t*-test; Figure 3C). The changes in the COX-2 and TNF-α mRNA levels corresponded to the changes in the levels of these proteins observed in our previous study [27]. Glucocorticoid-induced leucine zipper (GILZ, gene name: *Tsc22d3*), interleukin-1 receptor-associated kinases M (IRAKM, gene name: *Irak3*) and mitogen-activated protein kinase phosphatase 1 (MKP1, gene name: *Dusp1*) are anti-inflammatory genes that are transcribed following glucocorticoid receptor activation [28,29,30]. The mRNA levels of GILZ and IRAKM were significantly downregulated by oral ulcerative mucositis (*p* < 0.01 and *p* < 0.05, respectively, compared with the naive rats, Student’s *t*-test; Figure 3D,E), while the MKP1 mRNA level was not changed (Figure 3F).

Local treatment with Traful alone (*n* = 5) did not affect the expression of these genes compared with that of the non-treatment group (Figure 3A–F). However, the addition of Tmc to Traful (*n* = 5) downregulated the expression of inflammatory genes of COX-2 and TNF-α (*p* < 0.01 and *p* < 0.05, respectively) compared with that of the non-treatment group (Sidak’s test following one-way ANOVA; Figure 3A,C) and upregulated the expression of anti-inflammatory genes of GILZ, IRAKM, and MKP1 (*p* < 0.01 for all, Sidak’s test; Figure 3D–F), except for mPGES1 mRNA expression (Figure 3B). Although Tmc-containing Plastibase downregulated COX-2 mRNA expression and upregulated GILZ mRNA expression (*n* = 5, *p* < 0.01 and *p* < 0.05, respectively, Sidak’s test), the efficacies were weaker than those of Tmc-containing Traful (Figure 3A–F).

### 2.4. Anti-Allodynic Mechanism of a Steroid Containing Ointment in OUM Rats

**Figure 4:**
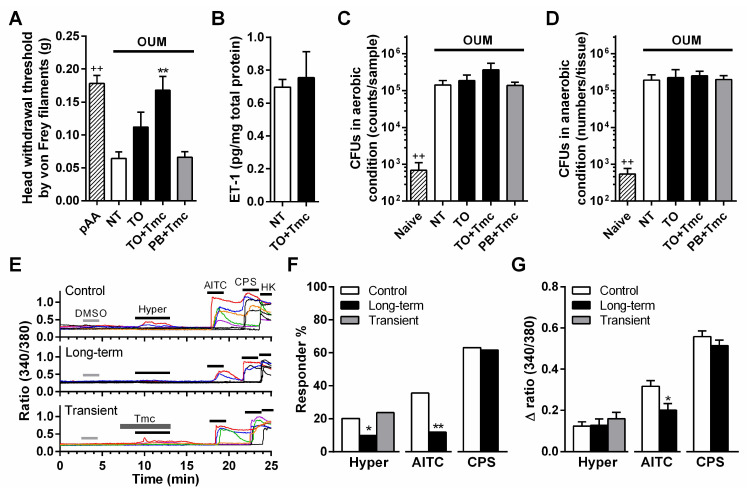
Analgesic effect of triamcinolone acetonide (Tmc)-containing Traful ointment (TO) on oral ulcerative mucositis-induced mechanical allodynia and its cellular mechanism. (**A**) Effects of ointments on oral ulcerative mucositis-induced mechanical allodynia. NT: non-treated oral ulcerative mucositis model (OUM), (pAA: before the acetic acid procedure in the same rats), TO: TO alone, TO + Tmc: TO with Tmc and PB + Tmc: Plastibase with Tmc; each group *n* = 5. ^++^ *p* < 0.01 between pAA and NT according to the Student’s *t*-test, *d* = 4.02. Among OUM rats, ** *p* < 0.01, compared with NT according to Sidak’s test following one-way ANOVA, *f* = 0.24. (**B**) Endothelin-1 (ET-1) levels in the oral ulcerative mucositis in the NT and TO + Tmc rats (each group *n* = 4, Student’s *t*-test, *d* = 0.25). Colony-forming units (CFUs) under aerobic (**C**) and anaerobic (**D**) conditions. Samples were collected from the oral ulcerative mucositis of the OUM rats two days after the acetic acid procedure. ^++^ *p* < 0.01 between the naive and NT groups according to Mann–Whitney U tests, *d* = 1.60, 1.35, respectively. The OUM rats were compared by one-way ANOVA, *f* = 0.47 and 0.77 (each group *n* = 5). (**E**) Representative Ca^2+^ responses to the hypertonic solution (Hyper, +100 mOsm), the TRPA1 agonist allyl isothiocyanate (AITC, 1 mM), the TRPV1 agonist capsaicin (CPS, 1 µM), and KCl solution (HK, 50 mM) in dissociated trigeminal ganglion neurons after treatments with Tmc (long-term: 3 h of incubation prior to the recordings; transient: 2 min incubation prior to hypertonic stimulation). DMSO application did not show any responses. (**F**) Percent responder to Hyper, AITC, and CPS. In the control, 20.1% (*n* = 30/149), 35.6% (*n* = 53/149), and 63.1% (*n* = 94/149) of the neurons were hypersensitive, AITC sensitive, and CPS sensitive, respectively. In the long-term incubation, 9.8% (*n* = 14/143), 11.9% (*n* = 17/143), and 61.5% (*n* = 88/143) of the neurons were hypersensitive, AITC sensitive, and CPS sensitive, respectively. In transient incubation, 23.6% of the neurons were hypersensitive neurons (*n* = 30/127). * *p* < 0.05 and ** *p* < 0.01, compared with the control according to Fisher’s test. (**G**) Mean *∆* ratio changes in Ca^2+^ responses. * *p* < 0.05 compared with the control according to Student’s *t*-test.

The head withdrawal threshold as assessed by von Frey filaments was significantly decreased at day 2 compared with that before the acetic acid procedure (*n* = 5, *p* < 0.01, Student’s *t*-test; Figure 4A), thus indicating the development of mechanical allodynia. Two coats of Tmc-containing Traful inhibited mechanical allodynia (*n* = 5, *p* < 0.01, compared with the non-treatment group, Sidak’s test following one-way ANOVA), but the application of Traful alone and Tmc-containing Plastibase did not inhibit this symptom (each *n* = 5; Figure 4A).

Treatment with Tmc-containing Traful did not change the endothelin-1 level or the number of colony-forming units (CFUs) under aerobic and anaerobic conditions on day 2 in the model group compared with the non-treatment group (Figure 4B–D). Endothelin and bacterial toxins sensitize mechanosensitive TRPA1 channels in nociceptive nerves [16,31]. Our recent study reported that the hypertonic stimulation-induced Ca^2+^ response in dissociated trigeminal ganglion neurons was caused by TRPA1 activation [32]. Therefore, we investigated the effect of Tmc on the hypertonic stimulation-induced Ca^2+^ response in sensory neurons (Figure 4E). The percent response to hypertonic stimulation and the TRPA1 agonist allyl isothiocyanate (AITC) was reduced to a lesser extent in the long-term preincubation group (3 h) than in the control group (*p* < 0.01 and *p* < 0.05, respectively, Fisher’s test; Figure 4F). The peak Ca^2+^ response induced by AITC was lower in the long-term incubation group than in the control group (*p* < 0.05, Student’s *t*-test), while that induced by hypertonic stimulation was not changed (Figure 4G). These inhibitory effects were not induced in the TRPV1 agonist capsaicin-induced Ca^2+^ response (Figure 4E–G). Transient preincubation for 2 min before hypertonic stimulation did not result in any changes (Figure 4E–G).

## 3. Discussion

The present study first supplies direct evidence of the molecular mechanism underlying the analgesic action of steroid ointments in oral ulcerative mucositis. Treatment with Tmc-containing Traful ointment suppressed both COX-dependent spontaneous pain and COX-independent mechanical allodynia in the rat model for oral ulcerative mucositis. The oral ointment base Plastibase, which is commonly used for human patients, had a residual period in rats that was insufficient to exert effective steroid actions. Therefore, experimental conditions using long-lasting ointments, Traful and PRO-quick, can be used to re-examine some early animal studies that failed to detect the efficacy of steroid ointments or active ingredients and will lead to new and more effective drugs for oral mucosal diseases.

Tmc- and dexamethasone-containing Traful inhibited spontaneous pain in the OUM. The pain modality has been reported to be a COX-dependent pathway [16,27]. Local treatment with Tmc-containing ointment inhibited prostaglandin E_2_ levels by suppressing COX-2 and TNF-α mRNA expression via upregulation of the expression of the glucocorticoid receptor-targeted anti-inflammatory genes of GILZ, IRAKM, and MKP1. Since mPGES1 mRNA was not changed by Tmc treatment, the enzyme was not related to the Tmc-induced inhibition of prostaglandin E_2_. Similar gene expression modulation was demonstrated in an early study that systemically administered dexamethasone [33]. Hence, the analgesic effect of steroids on oral ulcerative mucositis-induced spontaneous pain is elicited by well-known glucocorticoid receptor activation.

In contrast to spontaneous pain, oral ulcerative mucositis-induced mechanical allodynia has been reported to be caused by COX-independent mechanically sensitive TRP channel pathways: (1) TRPV4 sensitization due to elastases from neutrophils, and (2) TRPA1 sensitization due to endothelin and/or bacterial toxins [16,27,34]. The former pathway might be inhibited by the coats of Tmc-containing Traful, as shown by the little inflammatory cell infiltration in histology (Figure 2A). Therefore, Tmc treatment might withdraw the TRPV4-mediated mechanical allodynia. Based on the results of endothelin-1 production and bacterial invasion assessments in the ulcer region, we examined the effect of Tmc on TRPA1 function in peripheral nerves downstream of the latter pathway. The mechanical sensitivity of TRPA1 is indirectly enhanced by endothelin-1 [16] and directly enhanced by the bacterial toxin lipopolysaccharide [31]. Our recent study demonstrated that TRPA1 contributes to the hypertonic stimulation-induced Ca^2+^ response in dissociated trigeminal ganglion neurons [32]. In the present study, hypertonic stimulation-induced and AITC-induced Ca^2+^ responses were suppressed by long-term preincubation with Tmc, suggesting that TRPA1 suppression following the long-term effect of Tmc on peripheral nerves inhibits oral ulcerative mucositis-induced mechanical allodynia. Glucocorticoids have been reported to have nongenomic effects that do not require the glucocorticoid receptor, unlike the well-known classic genomic pathway [35,36]. However, transient preincubation did not affect the hypertonic stimulation-induced Ca^2+^ response. Hence, the nongenomic action of steroids was not involved in the analgesic effect via TRPA1 suppression. Since dexamethasone has been reported to suppress TRPA1 mRNA expression and protein levels in human chondrocytes [37], this signaling route may lead to TRPA1 suppression in sensory neurons. Since TRPV1 activity did not show any change following the long-term incubation of Tmc, the effect of Tmc on the channel would not be concerned with the analgesic effect.

In several clinical studies, whether steroid ointments accelerate oral ulcerative mucositis healing in patients with recurrent aphthous stomatitis has been debated [17,18,19,20,21]. Our results support that Tmc treatment did not cause delayed wound healing, it even had an analgesic effect. On day 2, the treatment suppressed inflammatory cell infiltration into the oral mucosa, but ulceration remained. Oral bacterial invasion through the ulcer might override steroid-induced wound healing. Suppression of TRPA1 activity by steroids may partly be associated with the loss of healing since a recent study reported that TRPA1 activation accelerates skin regeneration [38].

In conclusion, the representative steroid Tmc suppresses oral ulcerative mucositis-induced spontaneous pain by general anti-inflammatory actions following glucocorticoid receptor activation and mechanical allodynia by inhibiting mechanically sensitive TRPA1 in peripheral nerves. Long-lasting ointments in the oral mucosa such as Traful are needed as a superior drug delivery system to sufficiently elicit the anti-inflammatory and analgesic effects. Since there are sex differences in nociception and pain sensation [39], female rats should be further investigated to clearly establish the analgesic mechanism of steroids. Our established experimental conditions using Traful are useful to develop more effective locally administered drugs for oral ulcerative mucositis and periodontal diseases.

## 4. Materials and Methods

### 4.1. Ointments

The physical properties of Vaseline (Katayama Chemical Industries, Osaka, Japan), Plastibase (Taisho Pharmaceutical, Tokyo, Japan), TRAFUL^®^ base, and TRAFUL^®^ PRO-quick base (referred to as Traful and PRO-quick, respectively, in the present study; Daiichi Sankyo Healthcare, Tokyo, Japan) were assessed by a rheometer (Rheometer MCR302; Anton Paar, Graz, Germany), and their residual periods on the oral mucosa of human volunteers and rats were investigated. For animal experiments, Tmc or dexamethasone was kneaded into Plastibase or Traful.

### 4.2. Human Experiments

Students and faculties at Kyushu Dental University with no medical conditions and no oral ulcerative mucositis were recruited as healthy volunteers (*n* = 14, male: five, female: nine, 25.0 ± 8.03 years old). This human study was approved by the Medical Ethics Committee of Kyushu Dental University (approval no. 18–11). Informed consent was obtained from all the subjects prior to the experiment. A constant volume (50 mg) of the four kinds of ointment bases was randomly applied to four different areas (the back of the upper and lower lip) by the volunteer’s fingers. Immediately after the application, the subjects were instructed to score the ointments with the highest residual sensations as 10. For 2 h, the subjects were not allowed to drink or eat, and residual sensations of the ointments were repeatedly scored at 0.5, 1, and 2 h on a 10-point scale relative to the initial maximum sensation. After 2 h, an observer (M.N.) collected the residual ointments from the four application areas using plastic spatulas and weighed the collected residual ointments.

### 4.3. Animal Experiments

Male Wistar rats (*n* = 109, 8–10 weeks old; CLEA Japan, Inc., Tokyo, Japan) were used for all animal experiments. The rats were housed in pairs and maintained on a light–dark cycle (L:D, 12:12 h) under specific pathogen-free conditions with controlled humidity and temperature (40–60% and 21–23 °C, respectively). Rats were allowed to access water and food pellets ad libitum. All efforts were made to minimize animal suffering. All experiments were performed in accordance with the International Guiding Principles for Biomedical Research Involving Animals and were approved by the Animal Experiment Committee of Kyushu Dental University (approved no. 18-009 and no. 19-014). This study conformed with the ARRIVE (Animal Research: Reporting In Vivo Experiments) guidelines for preclinical animal studies.

As described previously [15], 50% acetic acid was used to induce oral ulcerative mucositis (*n* = 88). Naive rats including model rats prior to acetic acid treatments were prepared to confirm model establishment. The rats were randomly assigned to each experimental group regardless of litter and cages.

For the therapeutic treatment of rats with oral ulcerative mucositis, ointments (20 mg) were applied to oral ulcerative mucositis in conscious rats twice a day by using a toothpick (7:00–10:00 and 13:00–16:00). Rats without coating ointments (non-treatment group) were used as controls. K.O. was aware of the group allocation.

### 4.4. Rat Model of Oral Ulcerative Mucositis

Rats were anesthetized with intraperitoneal administration of a combination of three anesthetics (0.375 mg/kg medetomidine, 2.0 mg/kg midazolam, and 2.5 mg/kg butorphanol). Subsequently, a small filter paper (9 mm^2^) soaked in 50% acetic acid was placed on the labial fornix region of the inferior incisors for 30 s (*n* = 88). In the evaluation of the residual period in rats, ointment bases were applied to the labial fornix region of the inferior incisors of conscious rats. After 0.5, 1, and 2 h, the rats were anesthetized with 2% isoflurane, and the collected residual ointments were weighed. The measurements were performed repeatedly in the same rats (*n* = 4).

### 4.5. Evaluation of Oral Ulcerative Mucositis Severity

To evaluate the severity of oral ulcerative mucositis in the OUM on days 2–6, we used the following visual oral ulcerative mucositis score, which was modified from a previously reported score for the OUM [27]: 0, normal; 0.5, presence/absence of redness unclear; 1, slight but definite redness; 2, severe redness; 3, focal pseudomembrane, without a break in the epithelium; 4, broad pseudomembrane, with a break in the epithelium within the acetic acid-treated mucosal area; 5, virtual loss of epithelial and keratinized layers over the acetic acid-treated mucosal area; and 6, severe swelling of the lower lip with oral ulcerative mucositis at a score of 5.

### 4.6. Measurement of Blood Glucose Levels

Blood samples were collected from the tail vein of rats. The blood glucose level was measured by GLUCOCARD diameter-α (ARKRAY, Kyoto, Japan) on days 2–5 to confirm the effects of steroid ointment on blood glucose levels. The rats were fasted for 3 h prior to glucose measurement to eliminate the effects of food consumption on the blood glucose levels. As a control, the initial value at two days prior to acetic acid treatment was used.

### 4.7. Histology

Under deep anesthesia with a combination of anesthetics (0.375 mg/kg medetomidine, 2.0 mg/kg midazolam, and 2.5 mg/kg butorphanol), the lower lips of the naive and OUM rats were removed two days after the two ointment applications. Tissues were fixed overnight at 4 °C in 4% paraformaldehyde. After paraffin embedding, 4-µm-thick sagittal sections of the oral mucosa were stained with hematoxylin and eosin to investigate inflammatory cell infiltration. For histopathologic analysis, inflammatory aspects were evaluated by two independent observers in a blind manner. According to an early study [40], the following inflammatory score, which was simply modified to fit to the present study, was used for microphotographs (three sections at 12–16 µm interval per rats): Score 1, normal epithelium and connective tissue; Score 2, mild inflammatory cell infiltration; Score 3, moderate inflammatory cell infiltration; and Score 4, severe inflammatory cell infiltration, with representative guide microphotographs.

### 4.8. Enzyme-linked Immunosorbent Assays (ELISAs)

The mucosal tissue in the labial lips of the OUM rats (day 2) was extracted 2 h after the ointment treatments under deep anesthesia with the combined anesthetics. The tissues were homogenized in PBS with 10 µM indomethacin and a protease inhibitor cocktail. After centrifugation, the total protein concentrations of the supernatants were measured using a BCA Protein Assay Kit (Thermo Fisher Scientific, Waltham, MA, USA). According to the manufacturer’s instructions, the protein concentrations of endothelin-1 and prostaglandin E_2_ were measured with the following ELISA kits: Endothelin-1 Assay Kit (17165; Immuno-Biological Laboratories, Fujioka, Japan) and Prostaglandin E_2_ ELISA Kit (ab133021; Abcam, Cambridge, UK). The assays were carried out in duplicate. The concentrations were standardized to the total protein concentrations.

### 4.9. Bacterial Counts

All procedures were performed in a sterile environment. Under deep anesthesia, oral ulcerative mucositis tissues were collected from the lower lip of the naive and OUM rats (day 2) 2 h after the ointment treatments. The superficial skin layer was carefully removed before removing the lower lip. The tissues were cut into a small block (3 mm × 3 mm, 1–2 mm thickness) and chopped and sonicated in PBS solution in a 1.5 mL plastic tube for 30 s (38 kHz, US-2; SND). Fifty microliters of the suspension containing bacteria was plated in duplicate onto brain-heart infusion agar (Nissui Pharmaceutical Co. Ltd., Tokyo, Japan) without exceeding 500 counts per 90 mm diameter dish. The bacteria were cultured by overnight incubation at 37 °C. Anaerobic incubation was performed in an airtight container with AnaeroPack-Anaero, an O_2_-absorbing and CO_2_-generating agent (Mitsubishi Gas Chemical Co, Saga, Japan), followed by overnight incubation at 37 °C. The colony-forming units of the bacterial culture plates were manually counted.

### 4.10. Quantitative Reserve-Transcription Polymerase Chain Reaction

Mucosal tissues were extracted from the naive and OUM rats (day 2) 2 h after ointment treatments under deep combination anesthesia, immediately transferred to TRIzol^®^ Reagent (Thermo Fisher Scientific) and frozen at −80 °C. After homogenization, total RNA was isolated using a RNeasy Mini Kit (Qiagen, Hilden, Germany) and reverse-transcribed into cDNA by Superscript IV (Thermo Fisher Scientific) with random hexamers (Thermo Fisher Scientific). Two replicates from five rats were assessed for each group. cDNA was amplified in QuantStudio3/QuantStudio5 (Thermo Fisher Scientific) with PowerUp-TM SYBR^®^ Green Master Mix (Thermo Fisher Scientific) according to the manufacturer’s instructions. The relative gene expression levels were calculated by the *ΔΔ*Ct method. In addition to the primer sets for β-actin reported in our previous study [34], the following primer sets were used: *TNF* (TNF-α), 5′-TGA ACT TCG GGG TGA TCG-3′ and 5′-GGG CTT GTC ACT CGA GTT TT-3′; *Ptgs2* (COX-2), 5′-TAC ACC AGG GCC CTT CCT-3′ and 5′-TCC AGA ACT TCT TTT GAA TCA GG-3′; *Ptges* (mPGES1), 5′-CGT GAC TGG TGT GTG TCT CA-3′ and 5′-CTG CCC TCC TCT GAA AAC AT-3′; *Irak3* (IRAKM), 5′-CTG GAT AGC TGC GAT GGT C-3′ and 5′-CCA GCC AGC TGT TTG AAA GT-3′; *Tsc22d3* (also known as TSC22 domain family member 3, GILZ), 5′-GGG ATG TGG TTT CCG TTA AA-3′ and 5′-TTG TTG TCT AGG GCC ACC A-3′; *Dusp1* (MKP1), 5′-GTG CCT GAC AGT GCA GAA TC-3′ and 5′-CCA GGT ACA GGA AGG ACA GG-3′.

### 4.11. Pain Evaluations

To evaluate spontaneous pain, we measured mouth rubbing behavior with both forelimbs for 10 min in a clear plastic cage (30 × 30 × 30 cm) at a constant time (15:00–17:00) [15,16]. The measurements were conducted two days after the acetic acid treatments and 2 h after the ointment applications. Prior to this measurement, all rats were acclimated to the experimental conditions for three days (10 min per day).

The stable intraoral opening method described previously [15] was used to evaluate withdrawal mechanical thresholds in the oral mucosa of conscious rats. Under anesthesia, a magnetized needle (22-gauge-like size; Daiso Sangyo, Hiroshima, Japan) was pierced into the labial fornix region of the inferior incisors. The needle was then bent into a square ring, and the tip of the needle was removed. One week after piercing, the rats were trained to remain stable in a handmade restrainer (6 × 6 × 13 cm) and protrude their perioral region from the end of the restrainer. The training continued until the rats were able to perform the behavior smoothly (10 min per day for approximately 3–4 days). A small neodymium magnet (3 mm in diameter; Wako, Osaka, Japan), which was attached to a 4 g weight, was attached to the pierced ring to apply a constant vertical force. The rats were acclimated to the experimental conditions for 2–3 weeks, and then, head withdrawal thresholds to mechanical stimulations were measured using von Frey filaments (0.02–0.4 g; North Coast Medical, Morgan Hill, CA, and 0.1, 0.12, 0.2, and 0.3 g handmade filaments). The thresholds were determined to be the minimum pressure required to evoke withdrawal behavior in at least three of the five tests. The measurements were performed five times at 3–4 min intervals, and the values were averaged after excluding the maximum and minimum values [16,34]. Of the 43 trained rats, nine rats that did not enter the handmade restrainer were excluded from the experiment.

### 4.12. Ca^2+^ Imaging of Trigeminal Ganglion Neurons

Bilateral trigeminal ganglions were removed from 5-week-old naive rats under combination anesthesia, minced and incubated for 1 h in Ca/Mg-free HBSS (Thermo Fisher Scientific) with 2 mg/mL collagenase (Wako) and 2 mg/mL dispase (Sanko, Tokyo, Japan). For isolation of neurons, digested cell suspensions were centrifuged with 30% Percoll (Sigma–Aldrich, Saint Louis, MO, USA). After washing, the cell pellet was resuspended in L-15 Lebovitz medium (Thermo Fisher Scientific) with a 0.1% antibiotic/antimycotic solution. The cells were plated on 35 mm glass bottom dishes coated with poly-d-lysine (MatTek Corp., Ashland, MA, USA). The dishes were incubated at 37 °C in a humidified chamber for 3 h.

Before recording, plated cells were incubated with Fura-2AM (10 mM, Dojindo, Kumamoto, Japan) at 37 °C for 30 min. The dishes were mounted on the stage of an inverted fluorescence microscope (IX71; Olympus, Tokyo, Japan) and washed with an isotonic solution (mM): 88 NaCl, 5 KCl, 1 MgCl_2_, 2.4 CaCl_2_, 10 HEPES, and 110 mannitol. The solution was adjusted to pH 7.4. Fura-2 was excited every 0.5 s by alternate illumination with 340 and 380 nm light using a Ca^2+^ imaging system (Hamamatsu Photonics, Hamamatsu, Japan). The following drugs were used: triamcinolone acetonide (Wako) at 100 µM, the TRPA1 agonist allyl isothiocyanate (Wako) at 1 mM, and the TRPV1 agonist capsaicin (Wako) at 1 µM. These drugs were diluted as stock solutions in dimethyl sulfoxide and diluted 100-fold before the experiments. As a hypertonic stimulation, 100 mM mannitol was added to the isotonic perfusion solution. For examination of the long- or short-term effects of Tmc on Ca^2+^ responses, 100 µM Tmc-containing solutions were added to the medium 3 h before recordings or immediately before hypertonic stimulation. To confirm whether the recorded cells were neurons, we applied a 50 mM KCl solution at the end of each recording. Ca^2+^ responses are expressed as a ratio (F340/380), and the recorded cells were deemed to be sensitive to drugs and hypertonic solution if the *Δ* ratio was more than 0.05 after application. Under each experimental condition, the number of dishes tested was determined to keep a roughly equivalent number of neurons analyzed. All Ca^2+^ responses are expressed as a ratio (F340/F380), and the recorded cells were deemed to be sensitive to drug/osmotic stimulation if the *Δ* ratio was more than 0.05 after application.

### 4.13. Statistical Analysis

Data are presented as the mean ± SEM (or SD in Table 1), and n represents the number of human volunteers, rats, and neurons tested. The sample size was fixed at 3–6 rats and determined according to our previous studies [15,16]. No rats were excluded from the data analysis. GraphPad Prism 6 software (version 6.07 for Windows, 2015, GraphPad Software, Inc., La Jolla, CA, USA) was used for all statistical analyses. The effect size in animal experiments was calculated by G*power (version 3.1.9.7 for windows, 1992, programmed by Franz Faul, University Kiel, Germany). A paired or unpaired Student’s *t*-test was used to compare differences between two different groups. For comparison of between-group differences in the number of CFUs, the Mann–Whitney U test or Kruskal–Wallis test was used. Following two-way and one-way ANOVA, Tukey’s and Sidak’s post hoc tests, respectively, were applied to analyze three or more groups. Fisher’s test was applied to compare responder % to hypertonic and drug stimulations in Ca^2+^ imaging. Significance was accepted at *p* < 0.05.

## Figures and Tables

**Table 1:** Physical properties of ointment bases.

	Vaseline	Plastibase	Traful	PRO-Quick
Adhesiveness (N)	2.2 ± 0.1	1.4 ± 0.1	2.9 ± 0.1	4.1 ± 0.1
Hardness (N)	1.1 ± 0.1	1.3 ± 0.0	2.9 ± 0.5	2.7 ± 0.4
Viscosity, 1/s = 1 (mPa·s)	12.6 ± 1.7	86.8 ± 1.9	163.9 ± 16.4	173.7 ± 16.8
Viscosity, 1/s = 10 (mPa·s)	1.3 ± 0.1	9.6 ± 0.4	17.4 ± 1.7	25.5 ± 3.5

Mean ± SD. Each ointment was measured three times by a rheometer (Rheometer MCR302, Anton Paar).

## Data Availability

Data are contained within the article.
